# Data supporting the role of Fyn in initiating myelination in the peripheral nervous system

**DOI:** 10.1016/j.dib.2016.03.096

**Published:** 2016-04-01

**Authors:** Yuki Miyamoto, Moe Tamano, Tomohiro Torii, Kazuko Kawahara, Kazuaki Nakamura, Akito Tanoue, Shuji Takada, Junji Yamauchi

**Affiliations:** a Department of Pharmacology, National Research Institute for Child Health and Development, Setagaya, Tokyo 157-8535, Japan; b Department of Systems BioMedicine, National Research Institute for Child Health and Development, Setagaya, Tokyo 157-8535, Japan; c Graduate School of Medical and Dental Sciences, Tokyo Medical and Dental University, Bunkyo, Tokyo 113-8510, Japan

**Keywords:** Fyn, Myelination, Peripheral nervous system, Myelin marker protein, Signaling

## Abstract

Transgenic mice, which express active Fyn tyrosine kinase under the control of a glial fibrillary acidic protein promoter, have been produced. This promoter induces protein expression in the initiation stage of myelination in the peripheral nervous system (PNS) “Phosphorylation of cytohesin-1 by Fyn is required for initiation of myelination and the extent of myelination during development (Yamauchi et al., 2015 [1])”. Herein we provide the data regarding myelination-related protein markers and myelin ultrastructure in transgenic mice.

**Specifications table**

**Table:** 

Subject area	Biology
More specific subject area	Neurobiology, molecular and cellular neuroscience, developmental biology
Type of data	Figure
How data was acquired	Electron microscopy, immunoblotting
Data format	Raw data, analyzed data
Experimental factors	*g*-Ratios (numerical ratios of axon diameter to diameter of the axon׳s outermost myelinated fibers) for analyzing myelin thickness
Experimental features	Electron microscopic analysis, immunoblot
Data source location	National Research Institute for Child Health and Development, Tokyo, Japan
Data accessibility	Data is available with this article

## Value of the data

• This data set is of value to the scientific community to need the information for molecules controlling myelination.
• The data can provide the method of studying the initiation of myelination *in vivo*.
• The data may promote further research on signaling molecules controlling myelination *in vivo*.

## 1 Data

The data shared in this article is the biochemical analysis for myelination-related proteins in active Fyn transgenic mice. The data also provides myelin ultrastructure in transgenic mice.

## 2 Experimental design, materials and methods

We generated transgenic mice expressing active Fyn at the relevant developmental stage. The sciatic nerves of these mice were then analyzed through electron microscopy at 3 days postnatal and through immunoblotting of proteins such as myelin markers.

### 2.1 Data from Fyn transgenic mice

**Fig. 1:**
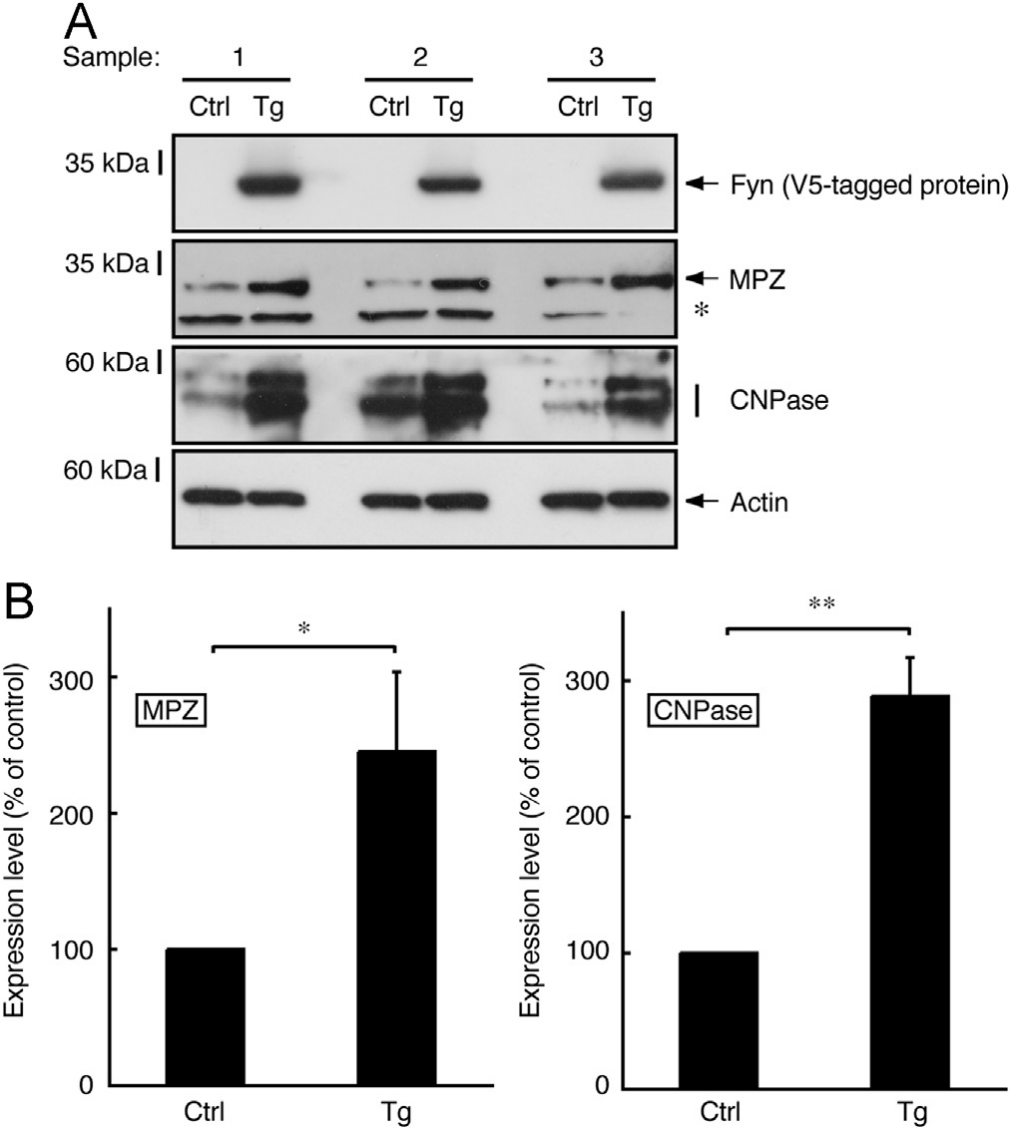
Increased expression of myelin marker proteins MPZ and CNPase in transgenic mice expressing active Fyn. (A) Tissue lysates (*n*=3 mouse samples) from 3-day-old sciatic nerves of active Fyn transgenic (Tg) and control (Ctrl) mice were used for immunoblotting with an anti-V5 tag (for V5-tagged active Fyn), MPZ, CNPase, or actin antibody. Control actin proteins are also shown. The positions of non-specific bands are indicated by an asterisk. (B) The scanned bands (MPZ and CNPase blots) were densitometrically analyzed for quantification. Data were evaluated using Student׳s *t*-test (**p<0.01; *p<0.05; *n*=3).

**Fig. 2:**
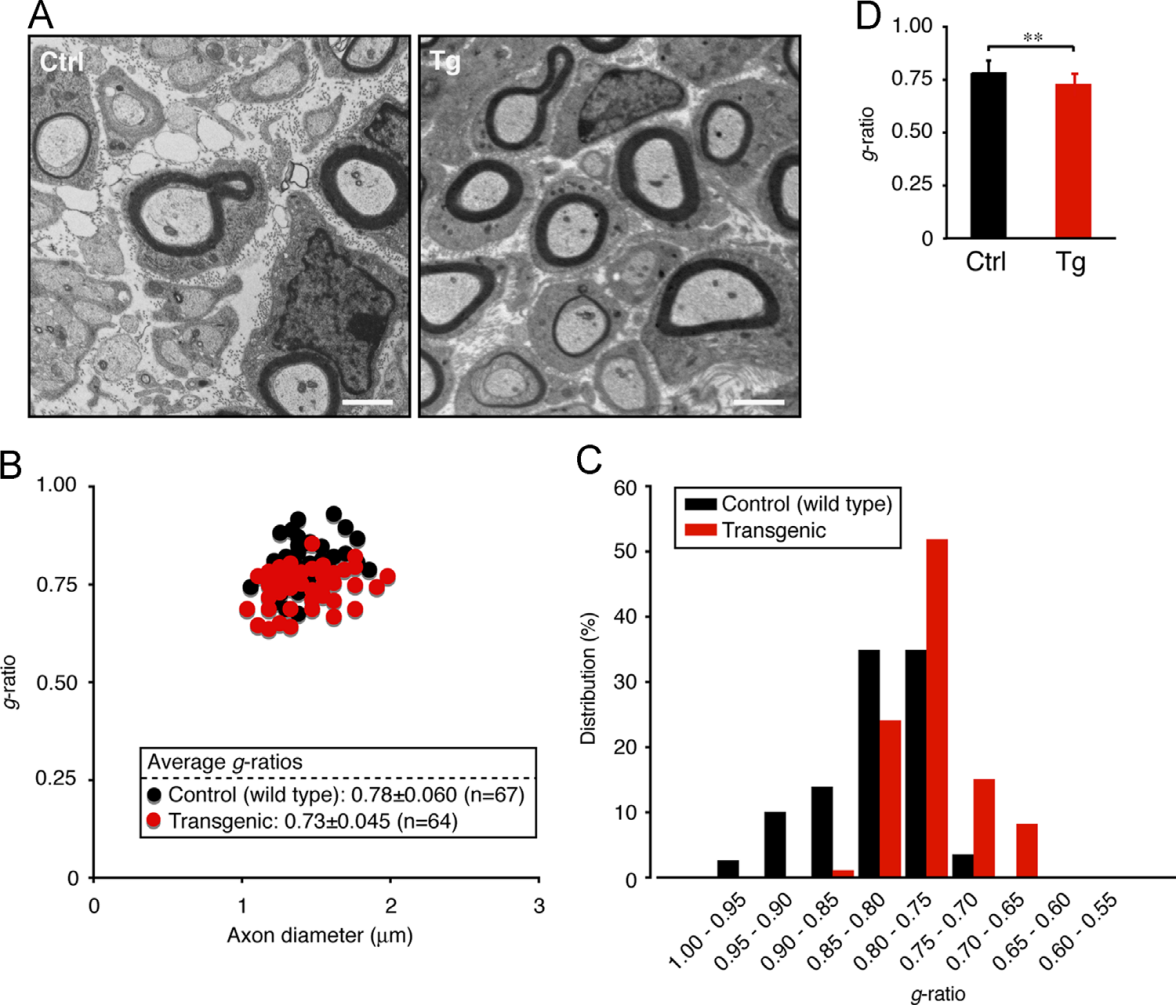
Transgenic mice exhibit increased myelin thickness. (A) Representative electron micrographs of 3-day-old transgenic (Tg) or control (Ctrl) mouse sciatic nerve cross sections are shown. The scale bars indicate 1 μm. (B) The *g*-ratios for 3 mice are plotted for axon diameters. The average *g*-ratios are also shown in the graph. (C) Distribution of the *g*-ratios is shown for axon diameters. (D) The *g*-ratios (*n*=67 control wild type mouse nerves and *n*=64 transgenic mouse nerves) were evaluated using Student׳s *t*-test (**p<0.01).

**Fig. 3:**
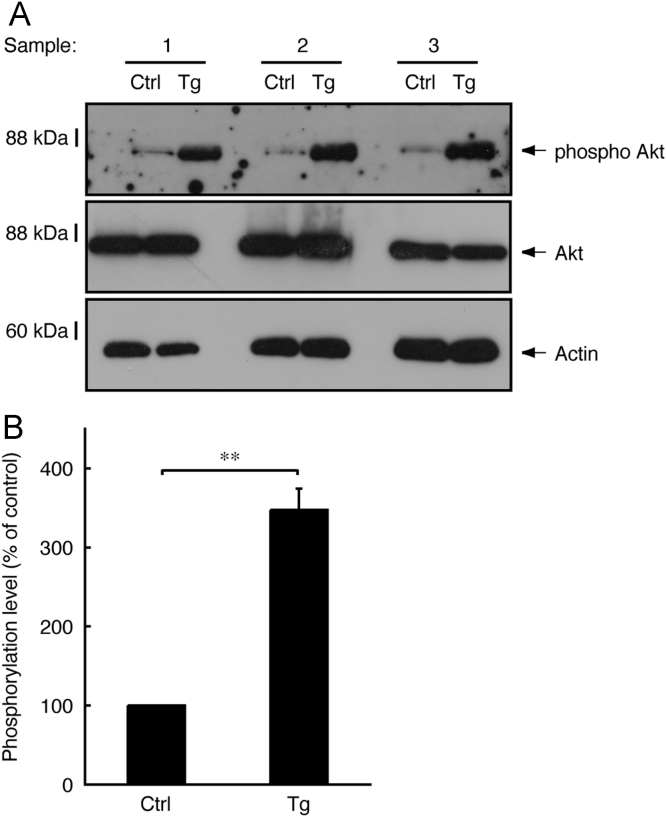
Elevated phosphorylation of Akt in transgenic mice. (A) Tissue lysates (*n*=3) from 3-day-old sciatic nerves of transgenic (Tg) and control (Ctrl) mice were used for immunoblotting with an anti-phosphorylated pan-Akt, pan-Akt, or actin antibody. (B) The scanned bands were densitometrically analyzed for quantification. Data were evaluated using Student׳s t*-t*est (**p<0.01; *n*=3).

**Fig. 4:**
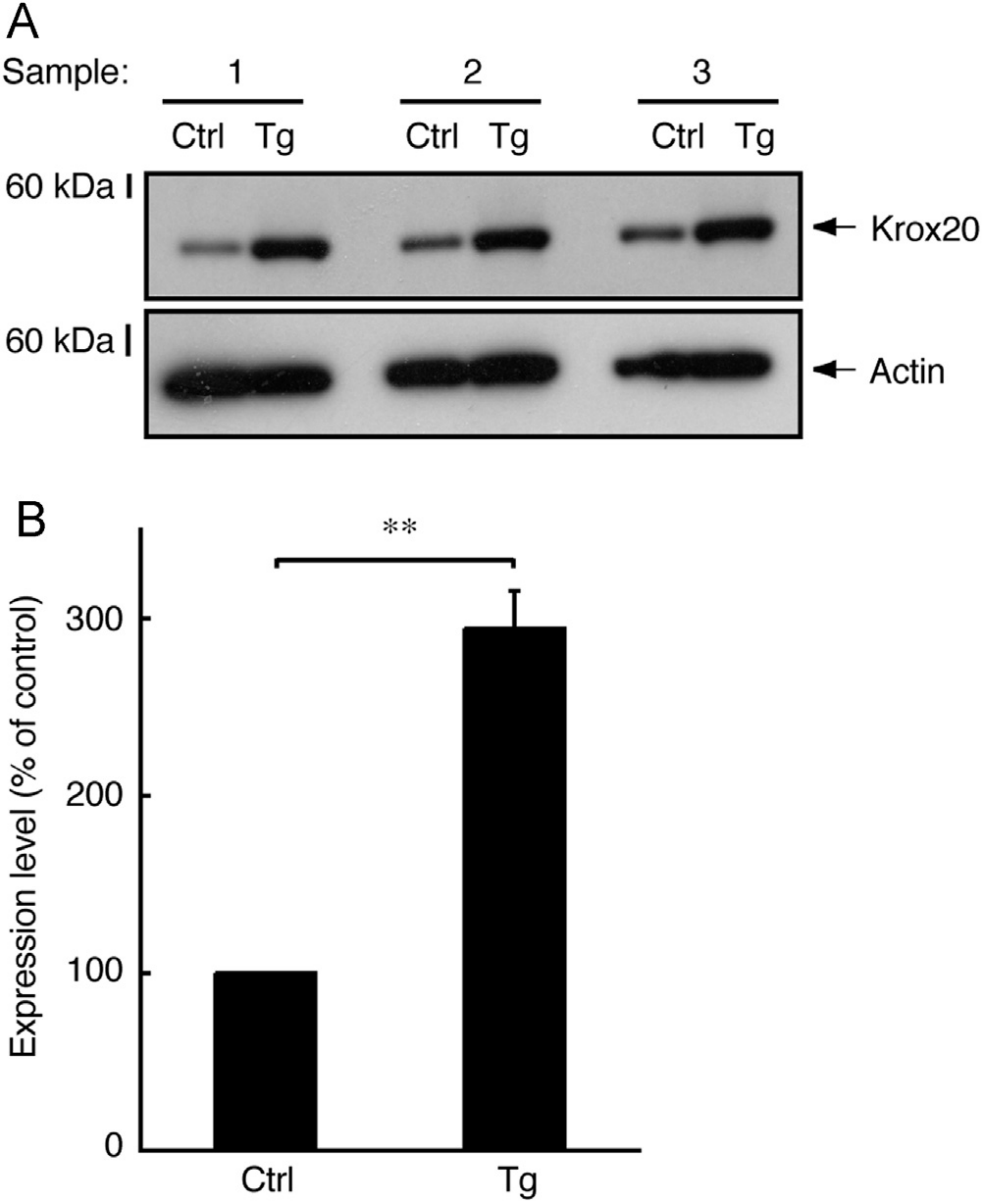
Increased expression of Krox20 in transgenic mice. (A) Tissue lysates (*n*=3) from 3-day-old sciatic nerves of transgenic (Tg) and control (Ctrl) mice were used for immunoblotting with an anti-Krox20 or actin antibody. (B) The scanned bands were densitometrically analyzed for quantification. Data were evaluated using Student׳s *t*-test (**p<0.01; *n*=3).

**Fig. 5:**
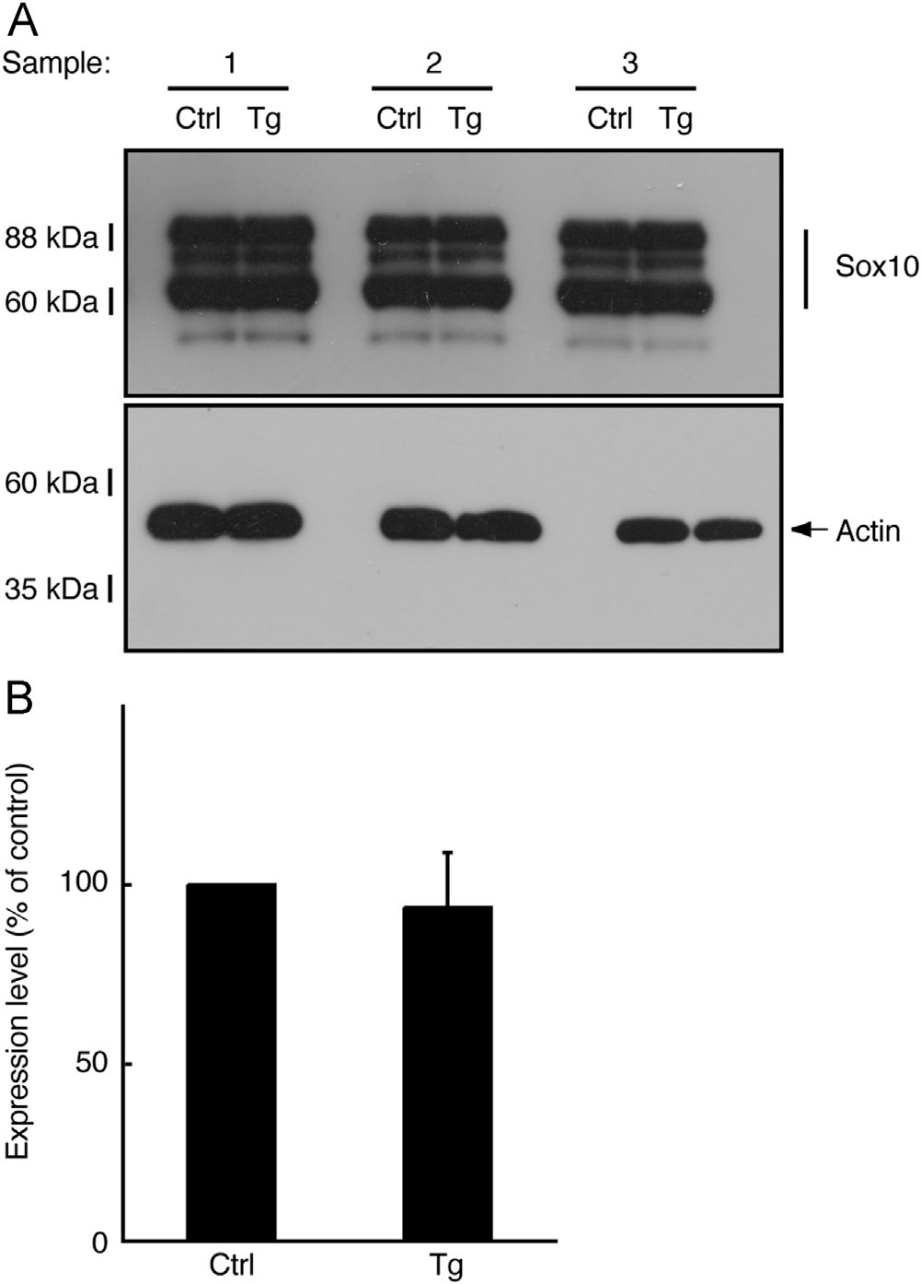
Expression of Sox10 was comparable in transgenic mice and controls. (A) Tissue lysates (*n*=3) from 3-day-old sciatic nerves of transgenic (Tg) and control (Ctrl) mice were used for immunoblotting with an anti-Sox10 or actin antibody. (B) The scanned bands were densitometrically analyzed for quantification.

**Fig. 6:**
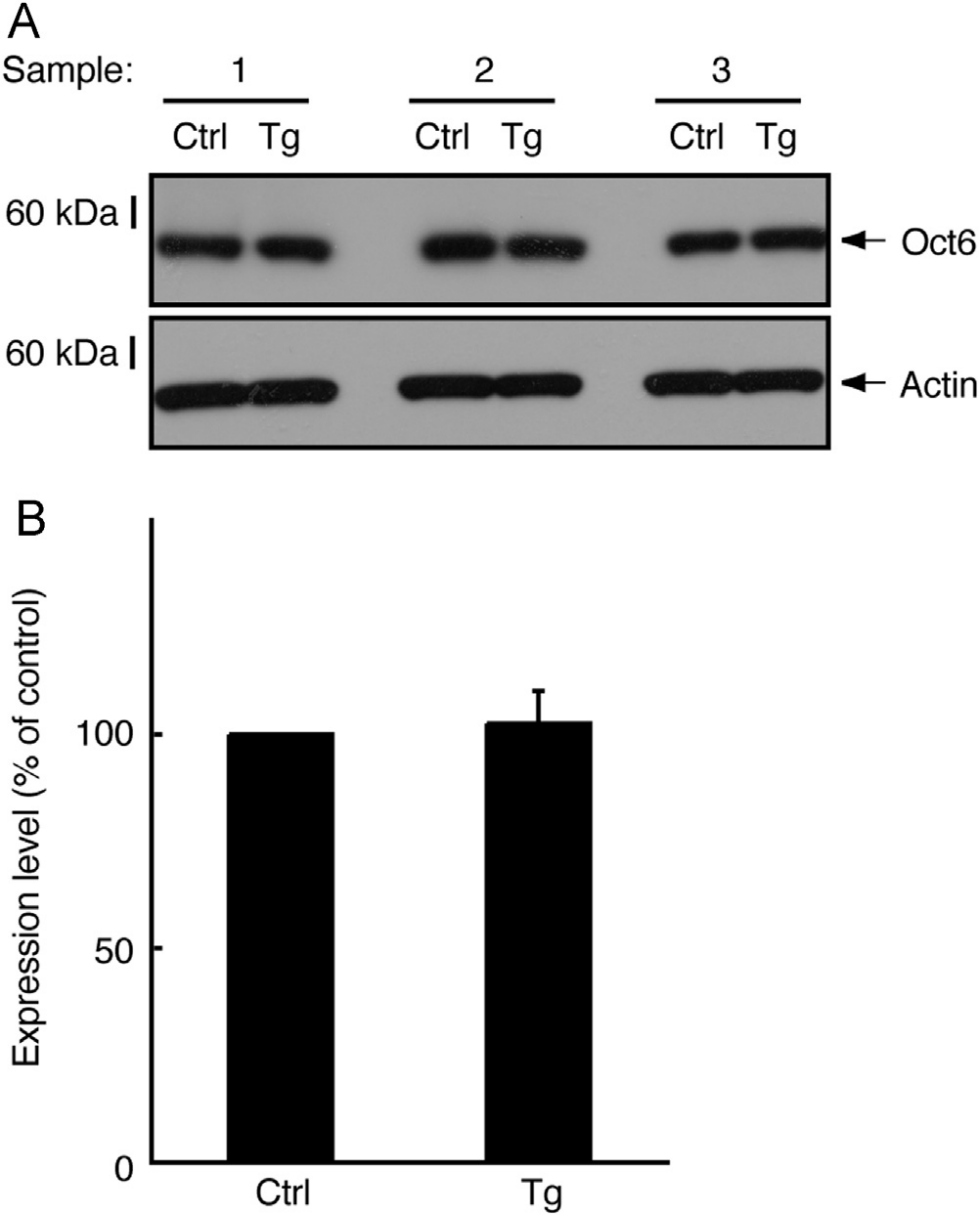
Expression of Oct6 was comparable in transgenic mice and controls. (A) Tissue lysates (*n*=3) from 3-day-old sciatic nerves of transgenic (Tg) and control (Ctrl) mice were used for immunoblotting with an anti-Oct6 or actin antibody. (B) The scanned bands were densitometrically analyzed for quantification.

In immunoblotting, neonatal transgenic mice expressing active Fyn exhibited increased expression levels of myelin marker proteins such as myelin protein zero (MPZ, also called P0) and 2′,3′-cyclic-nucleotide 3′-phosphodiesterase (CNPase) (Fig. 1A and B). In electron microscopic analysis, transgenic mice exhibited smaller *g*-ratios, indicating increased myelin thickness, in the sciatic nerves (0.73±0.045 in the transgenic mice compared to 0.78±0.060 in the control mice). Since the *g*-ratio is the numerical ratio of an axon׳s diameter to the diameter of the axon׳s outermost myelinated fibers [1], [2], [3], a smaller *g*-ratio indicates a thicker myelin sheath (Fig. 2A–D). In immunoblotting with an antibody specific for phosphorylated Akt kinase (active Akt), increased phosphorylation was observed in samples from transgenic mouse nerves (Fig. 3A and B). Akt is one of the central signal transducers controlling myelination [2], [3], [4], [5]. The myelination-associated transcription factor Krox20 [4], [5] was also increased in transgenic mouse nerves (Fig. 4, A and B). On the other hand, levels of Sox10 (Fig. 5A and B) and Oct6 (Fig. 6A and B), transcription factors expressed in Schwann cell lineage cells [4], [5], were comparable in transgenic mice and controls.

### 2.2 Generation of active Fyn transgenic mice

A DNA fragment (~4.5 kb) containing the SV40 enhancer, a mouse glial fibrillary acidic protein (GFAP) promoter specific for the neonatal stage of Schwann cells in the PNS [1], [6], [7], V5-epitope-tagged active Fyn (isolated Src homology domain 1 [1]), an artificial intron, and human chorionic gonadotropin polyA units [1], [7] was digested from the vector backbone (~3.5 kb) with NcoI, purified, and injected into fertilized BDF1 oocytes. Transgenic founder mice and established transgenic mice were routinely identified using the KAPA genomic PCR kit (KAPA Biosystems, Wilmington, MA, USA) with the specific primer pair 5′-CCGGAATTCGAATATTAGCTAGGAGTTTCAGAAAGGGGGCCTG-3′ and 5′-CCGGAATTCACTAGTGGGACTATGGTTGCTGACTAATTGAGATGC-3′. PCR was performed in 35 cycles, each consisting of denaturation at 94 °C for 0.5 min, annealing at 65 °C for 0.5 min, and extension at 0.5 °C for 1 min. The transgenic allele yielded PCR bands for 322 bases. One transgenic founder was obtained from every 240 fertilized oocyte injections. Transgenic founders were mated to wild type C57BL/6JJms mice. The transgene was stably maintained for at least 3 generations. Male mice were used for experiments when their gender was distinguishable.

### 2.3 Immunoblotting

Mouse sciatic nerves were lysed in lysis buffer (50 mM HEPES-NaOH, pH 7.5, 20 mM MgCl2, 150 mM NaCl, 1 mM dithiothreitol, 1 mM phenylmethane sulfonylfluoride, 1 μg/ml leupeptin, 1 mM EDTA, 1 mM Na_3_VO_4_, and 10 mM NaF) containing detergents (0.5% NP-40, 1% CHAPS, and 0.1% SDS). The presence of these detergents is important for myelin protein isolation [7], [8]. Equal amounts of the proteins (20 μg total proteins) in centrifuged cell supernatants were heat-denatured for immunoblotting using the MiniProtean TetraElectrophoresis and TransBlot TurboTransfer System (Bio-Rad, Hercules, CA, USA). The transferred membranes were blocked with the Blocking One kit (Nacalai Tesque, Kyoto, Japan) and immunoblotted using primary antibodies, followed by peroxidase-conjugated secondary antibodies (Nacalai Tesque). The bound antibodies were detected using the ImmunoStar Zeta kit (Wako, Osaka, Japan). The scanned bands were densitometrically analyzed for quantification using UN-SCAN-IT Gel software (Silk Scientific, Orem, UT, USA). The following antibodies were used: polyclonal anti-MPZ and monoclonal anti-actin from MBL (Aichi, Japan); polyclonal anti-CNPase, monoclonal anti-pan-Akt, and monoclonal phosphorylated pan-Akt (active, phosphorylated Ser-473) from Cell Signaling Technology (Danvers, MA, USA); anti-Krox20, anti-Oct6, and anti-Sox10 from Abcam (Cambridge, UK); and anti-V5 epitope from Nacalai Tesque.

### 2.4 Electron microscopic analysis

Mouse sciatic nerves were fixed with 2% paraformaldehyde and 2% glutaraldehyde in 0.1% cacodylate buffer [1], [7]. The tissues were postfixed with buffered 2% osmium tetroxide, dehydrated with an ethanol gradient, treated with acetone, and embedded in epoxy resin. Ultrathin sections of cross sections were stained with uranyl acetate and lead citrate, then observed and photographed with the Hitachi H-7600 or JEOL JEM-2010 electron microscope system. Myelinated nerves in the cross sections were randomly selected, and the *g*-ratio was calculated for each axon and as an average.

### 2.5 Statistical analysis

Data are presented as means±SD from independent experiments. Intergroup comparisons were performed using unpaired Student׳s *t* test. Differences were considered significant when *p* value was less than 0.05.

## Conflict of interest

The authors declare that there is no conflict of interest.
