# Supplementary material for: Data supporting the role of Fyn in initiating myelination in the peripheral nervous system
Source: Data Brief. 2016 Apr 1;7:1098–105. doi: 10.1016/j.dib.2016.03.096 (PMC4833127; doi:10.1016/j.dib.2016.03.096)
Supplement: Supplementary file 1 — Supplementary material [file mmc1.pdf]

***Conflict of Interest Form**

Click here to download Conflict of Interest Form: coi_disclosure11.pdf

***Conflict of Interest Form**

Click here to download Conflict of Interest Form: coi_disclosure12.pdf

*Conflict of Interest Form
Click here to download Conflict of Interest Form: coi_disclosure13.pdf

*Conflict of Interest Form

Click here to download Conflict of Interest Form: coi_disclosure14.pdf

*Conflict of Interest Form
Click here to download Conflict of Interest Form: coi_disclosure15.pdf

*Conflict of Interest Form
Click here to download Conflict of Interest Form: coi_disclosure16.pdf

*Conflict of Interest Form
Click here to download Conflict of Interest Form: coi_disclosure17.pdf

***Conflict of Interest Form**

Click here to download Conflict of Interest Form: coi_disclosure18.pdf
